# The perception of facilitators and barriers to the use of e-health solutions in Poland: a qualitative study

**DOI:** 10.1186/s12911-024-02791-x

**Published:** 2024-12-18

**Authors:** Paulina Smoła, Iwona Młoźniak, Monika Wojcieszko, Urszula Zwierczyk, Mateusz Kobryn, Elżbieta Rzepecka, Mariusz Duplaga

**Affiliations:** 1 Department of Health Promotion and e-Health, Faculty of Health Sciences, Institute of Public Health, Jagiellonian University Medical College, Skawińska Str. 20, Krakow, 31-066 Poland; 2 Department of Epidemiology and Population Studies, Faculty of Health Sciences, Institute of Public Health, Jagiellonian University Medical College, Skawińska Str. 20, Krakow, 31-066 Poland

**Keywords:** E-health, Telemedicine, In-depth interviews, Thematic analysis, Medical data sharing

## Abstract

**Background:**

E-health entails the use of information and communication technologies in support of health and health-related activities. E-health increased significantly during the COVID-19 pandemic in Poland. The pandemic showed that the e-health environment may be an important element of the response to epidemiological challenges. Polish citizens were provided with an array of e-health tools supporting the provision of health services.

**Methods:**

The main aim of the study was to assess the knowledge, use, and opinions about e-health solutions in Polish society. Fifty participants representing the general population took part in in-depth interviews. The interviews were conducted face-to-face with participants in their homes or via a teleconferencing platform from November 2023 to January 2024. At first, the interviewees were recruited by convenience, and at a later stage, a snowballing approach was applied. A semi-structured guide covered the knowledge about and use of e-health solutions, attitudes toward new technologies, and opinions about artificial intelligence and robots in healthcare. The interviewers interviewed 50 participants, of whom 26 were females. The interview transcriptions were analyzed with MAXQDA Analytics Pro 2022 (Release 22.7.0). An approach based on thematic analysis was employed to evaluate the interviews’ content.

**Results:**

Thematic analysis of the interviews resulted in the identification of three main themes: (1) knowledge about e-health, (2) barriers, and (3) facilitators of e-health use. Recognition of the term ‘e-health’ was limited among study participants, although they used e-health solutions frequently. The main barriers included limited digital skills and unfavorable attitudes to new technologies. Some of the participants complained about technical difficulties, e.g., poor Internet access. The main facilitators identified based on the interviews include saving time and reducing costs, as well as the ability to access medical records in one repository, as in the case of the Internet Patient Account. Some people believed e-health to be an element of progress. Overall, the study participants supported sharing their medical data for research.

**Conclusions:**

Implementing e-health solutions seems to be perceived as an inevitable consequence of technological progress. However, a lack of adequate technical skills remains one of the major obstacles to efficiently utilizing e-health’s potential.

**Supplementary Information:**

The online version contains supplementary material available at 10.1186/s12911-024-02791-x.

## Background

The use of telemedicine and e-health solutions increased significantly during the COVID-19 pandemic in many countries [1]. Preventive measures, especially social distancing, introduced in response to the threat of transmission of infection, propelled interest in the remote physician visits and services covered by the term ‘e-health.’ Before the pandemic, typical telemedicine services were rarely available in Poland. In 2016, only selected types of interactions (relating to second opinions), mainly in cardiology and geriatrics, were approved by the Agency of Health Assessment and Tariffication and reimbursed by the National Health Fund (NHF) [2]. The option to contact a physician remotely was not commonly available for patients in the public healthcare system due to the lack of a clear reimbursement scheme. In November 2019, just a few months before the pandemic, remote physician visits were introduced as an alternative to traditional visits [3]. According to a survey carried out in 2019, only 17.6% of respondents were able to define the term “telemedicine,” and only 14.4% of respondents declared the use of telemedicine-based services [4].

The COVID-19 pandemic marked a significant shift in the utilization of telemedicine and e-health services in Poland. The first case of COVID-19 was diagnosed on March 4, 2020, and shortly after, the Chief Sanitary Inspector recommended using remote (telephone-based or videoconferencing) visits to contact primary care physicians. A few days later, such services were also introduced to ambulatory specialist care. Remote physician visits were mainly conducted over the telephone, and only a very small portion of them were delivered via video-teleconference. A survey conducted under the auspices of the Ministry of Health and the NHF showed that during the first four months after the beginning of the pandemic in Poland, as many as 81.8% of primary care visits were carried out remotely, mainly by telephone [5]. Overall, only 4.4% of all episodes of primary and specialist ambulatory telemedicine were carried out on video-teleconferencing applications in the first months of the pandemic [6]. Another study showed that in the first six months of the pandemic, 57.6% of citizens who were internet users had benefited from some type of remote physician advice. Among other e-health applications, the use of e-prescriptions was declared by 56.1%, e-sick leaves by 16.6%, and e-referrals by 16.3% of respondents [7].

The abrupt introduction of remote visits to the public healthcare system was perceived as a type of natural experiment, as earlier, they had only been sporadically available. However, patients’ satisfaction, especially in the first phase of the pandemic, with such remote health services was very high. According to a survey in October 2020, as many as 85.4% of respondents stated that they had managed to resolve most of their issues thanks to a physician’s teleadvice [6]. Furthermore, 61.5% favored maintaining such services after the pandemic [6]. Such a level of satisfaction with primarily telephone-based remote visits was unexpected. Communicating medical problems in a telephone conversation may be difficult for a patient. Furthermore, for a physician, the telephone does not allow one to assess a patient’s status to the same degree as during a videoconference. Irrespective of these limitations, remote telephone-based visits became a popular option for contacting family physicians and, to some extent, specialists in the next years of the pandemic. The usage of remote visits and e-health services has remained common in the post-pandemic era in Poland [8], although the Ministry of Health introduced a set of restrictions to their use [9].

The World Health Organization has defined e-health as “the cost-effective and secure use of information and communication technologies (ICT) in support of health and health‐related fields” [10]. In turn, telemedicine is usually understood as the use of information and telecommunication technologies to deliver clinical care to patients. Thus, remote physician visits would be a typical form of telemedicine. The broad definition of e-health cited here means that telemedicine should be interpreted as a form of e-health. It should also be noted that such a broad definition of e-health is contested by some authors who consider telemedicine a key term for remote health services [11]. In this paper, we will adhere to the WHO’s definition of e-health, encompassing telemedicine as a special type of solution enabling remote contact between patients and physicians or, as in the case of so-called ‘second opinion’ interactions, between various physicians.

It seems that one of the main factors adding to the success of telephone physician visits was the supporting role of other e-health services implemented in the Polish healthcare system before the pandemic or shortly after it began. In 2018, widespread e-prescription pilot programs were completed, and this solution became obligatory for all physicians in January 2020 [3]. Since 2018, Polish physicians have been obliged to issue sick leave confirmations electronically [12]. In January 2021, e-referrals also became commonly available. In the first year, more than 62 mln. e-referrals were generated [13]. Apart from these three types of e-health services, many healthcare facilities have allowed their patients to make appointments for ambulatory care through electronic platforms for several years. Most biochemical and imaging laboratories also offer patients web-based access to diagnostic test results. A set of e-health solutions implemented at the national level by the E-health Centre, affiliated with the Ministry of Health, has increased the usefulness of remote telephone visits.

The first vaccines against COVID-19 became available in Poland at the beginning of 2021. Access to vaccination and support for related procedures like making appointments or obtaining vaccination certificates was hugely enhanced by using the Internet Patient Account, a portal available to all Polish citizens eligible for public health coverage [14]. The IPA was first implemented as a platform for people to track their interactions with the healthcare system. In the following years, more functionalities were gradually added. Currently, the IPA allows users to check e-prescriptions, e-referrals, history of obtained health services, and paid rates of health insurance. According to a study carried out in June 2023, the IPA portal had been used by 43% of Polish citizens [8].

The COVID-19 pandemic radically changed the landscape of healthcare services, and e‑health services, understood broadly and encompassing typical telemedicine encounters, became a common way of healthcare delivery in Poland. The widespread introduction of remote televisits in Poland was a reaction to the challenge of the pandemic threat, and citizens’ reactions were examined only retrospectively. Before the COVID-19 pandemic, there were only a few studies on potential user opinions of ehealth and telemedicine. Some of these were conducted during an era of an e-health-naive society [15]. Our study’s main aim was to analyze Polish citizens’ knowledge about the ehealth environment and their attitudes regarding the broad introduction of ehealth solutions. We have also analyzed their perception of the facilitators and barriers to using e-health solutions in Polish society. Finally, we have assessed the use of e-health solutions, including e-prescriptions and the Internet Patient Account portal. To achieve these aims within broader research initiative we have conducted a qualitative study based on in-depth interviews among representatives of general society.

## Methods

### Study design

The study was conducted as an element of a research project aimed at identifying the key determinants of using e-health solutions and Health 4.0 technologies in Polish society. We conducted a series of in-depth interviews based on an interview guide, focusing on various aspects of participants’ perceptions and use of e-health solutions. The main components of the interview guide included, apart from the knowledge and the use of ehealth applications, the attitude to new technologies and technology anxiety, the use of the internet to access health-related information, digital communications with other persons in relation to health issues, the experience with remote physician visits, the use of m-applications and the perception of own digital skills. The structure of the interview guide is available in Table S1. A pilot study was performed to ensure that the questions in the interview guide were understandable and pertinent to the participant’s situation. The pilot interviews confirmed the feasibility of the structure of the interview guide. However, they revealed that the language of interviews should be simplified, especially considering terms used to describe e-health solutions and technologies falling into the domain of health 4.0. It became obvious that the interviewees needed examples of e-health services to better understand the context of questions and express their opinions. Asking about personal experience in using specific services was also an important facilitator of a common understanding of issues addressed during interviews. In the stage of the pilot activities, no unexpected issues were signalled by the participants.

The obtained research material was transcribed and then analyzed using MAXQDA Analytics Pro 2024 software. The analysis of qualitative data was based on thematic analysis according to the recommendations of Braun and Clarke [16]. Such an approach allows the identification of gaps in the literature and the understanding of commonalities for a given topic.

The study was approved by the Bioethical Committee of the Jagiellonian University (Decision No 1072.6120.296.2022 issued on January 18, 2023). All interviewees received printed information about the study’s aims and methods. If needed, interviewers provided further explanations. Informed consent was obtained from every interviewee before the initiation of the interview. At the beginning of the interview, participants were asked if they would like to receive the report on the study’s results by e-mail.

The consent forms were numbered sequentially by interviewers. The files containing the original and revised recordings and transcriptions did not include the interviewees’ personal data.

### Data collection

The interviews took place between October 2023 and January 2024. The study participants were residents of the eastern-southern part of Poland, mainly of the Malopolskie and Swietokrzystkie Voivodships. They were recruited through snowballing sampling technique. The interviewers initiated interviews with individuals available at the time and place, e.g., inhabiting a specific location or attending healthcare facilities. In the next phase, they asked the first participants to share contacts with other potential participants willing to join the study. The research team strived to assure maximum diversity in terms of the sociodemographic profile of the study sample, especially regarding gender, age, education levels, and vocational status. Most of them were conducted face-to-face in the interviewees’ homes. About ten interviews were conducted via teleconference on the Microsoft Teams Classic platform. All interviews were recorded with portable voice recorders or the option available in the videoteleconferencing systems and transcribed by the interviewers. Each interview was conducted only once. At the end of each interview, a summary was prepared. The interviewee’s natural way of speaking was preserved during transcription. All grammatical errors, interruptions, repetitions, and the basic emotions of the interviewee in response to the questions, such as laughter or obvious fear of not knowing the answers, were considered. Transcripts were not returned to interviewees for feedback or correction. Interviewees were also not asked for their views on the research team’s findings but if they would like to read the articles based on the interview data. After publishing the relevant papers, the research team will contact those interested in the study’s findings. The interviewees’ quotes included in the article were corrected grammatically and stylistically, with repetitions and other irregularities removed. They were then translated into English.

Eleven people refused to participate in the survey. In four cases, the refusal was justified by a lack of time. Three persons refused to participate in the interview because they believed they didn’t know enough about e-health. For three potential interviewees, the recording of the interview and the security of personal data were reasons for refusal. One person did not give any reason for rejection.

### Quality criteria

The study was guided by the Consolidated Criteria for Reporting Qualitative Research (COREQ) [17]. The designer and coordinator of the study was a medical doctor. The interviews were conducted by a research team consisting of EB, UZ, MK, MW, and PS. The team included a historian/archivist working as a research assistant, a nutritionist, a sociologist, a nurse, and a public health specialist. To improve the researchers’ skills in qualitative methods, IM, a sociologist, conducted a series of workshops on qualitative methods to standardize the researchers’ knowledge. All research team members were employed in academic positions in the Department of Health Promotion and e-Health or were Ph.D. students in the School of Medicine and Health Sciences at Jagiellonian University Medical College. IM and PS carried out thematic analysis and coding. Compliance with COREQ requirements is described in Table S1, which is available in the Supplementary File.

### Data analysis

Thematic analysis was chosen because, according to Braun and Clarke, it allows us to explore the hidden meaning of the analyzed content and obtain a broader understanding of the research problem.

Thematic analysis was carried out following the six stages proposed by Braun and Clarke’s model [16, 18]:

1. Familiarization with the data
  Reading the research material before coding allowed us to get an overall picture of the data.
2. Generating initial codes
  Eight hundred forty-one codes were created on topics covering knowledge about e-health, the use of e-health in practice, facilitators and barriers to new technologies, and attitudes toward sharing medical data. The number of codes was gradually reduced after consultation with the coders.
3. Searching for themes
  Although this is distinguished as a separate step, searching for themes started when the coding process was advanced enough to recognize the patterns of relationships between codes. Here, similarly to the previous step, coders worked independently at first.
4. Reviewing potential themes
  After the coders independently defined initial themes, they confronted and compared their findings to find agreement.
5. Defining and naming themes
  At this step, the working titles of the themes were changed into analytical ones, and then their suitability and accuracy were checked to answer the research question.
6. Preparing the report

The codes were introduced inductively from the research material. The code tree contains inductive and deductive codes grouped thematically (Table S2). They cover the main research areas such as opinions about ehealth solutions, ‘experience with their use,’ ‘barriers to new technologies in health,’ and ‘facilitators to their use.’ They are complemented by sub-codes that specify interviewees’ statements in the context of research questions. For example, the code ‘barriers to new technologies in health’ is supplemented by the sub-code ‘personal data,’ which refers to the fact that for the interviewee, the main barrier to using new technologies is the fear of losing control of personal data.

## Results

### Participant characteristics

Interviewees were recruited for the study based on socio-demographic characteristics (gender, age, place of residence, education, occupational status) and technology use. Individuals with different socio-demographic characteristics were selected to verify the main barriers and ascertain the acceptance of new technologies and e-health. Each interviewee was interviewed only once. A snowball sampling technique was used for recruitment. After the first interviews were conducted, the research team discussed the introduction of possible modifications to the scenario. Ultimately, 50 interviews were conducted with 26 women and 24 men. The interviewees’ mean age (standard deviation, SD) was 43.8 (14.2). The age range of participants was from 18 to 76. The detailed characteristics of the study group are provided in Table 1.

**Table 1:** Characteristics of the study group

Variable	Categories of variable	%	*n*
**Age (years)**	18–29	18.0	9
	30–39	24.0	12
	40–49	14.0	7
	50–59	34.0	17
	> 59	10.0	5
**Sex**	Female	52.0	26
	Male	48.0	24
**Place of residence**	Rural	48.0	24
	Urban < 100,000 population	12.0	6
	Urban of 100.000–500,000 population	8.0	4
	Urban > 500,000 population	32.0	16
**Education**	Lower than secondary	28.0	14
	Secondary or post-secondary	48.0	24
	University degree	24.0	12
**Vocational status**	Public sector employee	76.0	38
	Retired	8.0	4
	University or college student	4.0	2
	Unemployed/Inactive	12.0	6
**Marital status**	Single	34.0	17
	Married	56.0	28
	Widowed/divorced/in separation	10.0	5
**Chronic disease**	No	88.0	44
	Yes	12.0	6
**State of health**	Excellent	14.0	7
	Very good	34.0	17
	Good or satisfactory	48.0	24
	Unsatisfactory	4.0	2
**Internet usage time per day**	No usage	6.0	3
	to 30 min.	6.0	3
	> 30 min. to 1 h	18.0	9
	> 1 h to 2 h	18.0	9
	> 2 h	52.0	26

### Themes

Four themes covering the e-health views and experiences of the interviewees were distinguished.

### Theme 1: knowledge and experience

The first theme establishes whether interviewees know the term ‘e-health,’ what they associate with it, and what their experience has been with health-related applications, especially the IPA.

A) Familiarity
  Not surprisingly, most of the interviewees did not know the term. An example of a typical response is given below:
  *Researcher*
  *Are you familiar with the term e-health?*
  *Interviewee*
  *This is, this is.... e-health is... I can't, I don't know. (MWoj_9_07/01/2023, Item 165-167)*
  Some interviewees associated the term ‘e-health’ with telemedicine.
  *Well, this medicine is teleconsultation, solving health problems over the phone with a doctor, for example, maybe this is e-health. Well, it's possible that this is some nutritional, dietary or sports advice. (EB_7_16/12/2023, Item 195*)
  Some interviewees were familiar with the word but did not know its exact meaning.
  *E-health? No, I mean, maybe I've heard it somewhere, but I don't remember anything specific about it. (MK_2_10.10.23, Poz. 124-126)*

B) Experience

It turned out that unfamiliarity with the term ‘e-health’ did not correlate with a lack of experience using e-health applications. Interviewees used e-health solutions from time to time, and some of them even regularly.

*Researcher: Do you use electronic services in health care? For example, e-referrals?*

*Interviewee: Yes, I use it, which means I get it. First of all, I have applications and when necessary, I use them.*

*Researcher: Do you register with a doctor online?*

*Interviewee: It happened a few times via e-patient.*

*Researcher: Do you use e-prescriptions?*

*Respondent: Yes*

*(EB_3_4/12/2023, Item 213-228)*

There are several aspects of using the most popular types of e-health applications:

a The role of remote physician visits and other e-health applications during the pandemic
  For most interviewees, the COVID-19 pandemic was the reason they started using ehealth solutions, mainly teleconsultations. The introduction of some applications coincided with the beginning of the pandemic, as in the case of e-prescriptions. Some interviewees saw such applications as a positive change in how they interacted with a doctor. They emphasized that they felt safer and more comfortable thanks to reducing the need to visit the clinic.
  *If there was a risk of infection*,* it was better not to see other people directly*,* including a doctor. (MK_1_8.10.23*,* Item 285)*
b Most frequently used e-health solutions
  Interviewees were most frequently familiar with e-prescriptions and less commonly with e-referrals and electronic appointments for physician visits. However, not every public clinic offers the option of e-registration yet. The use of e-referrals became obligatory in the second year of the pandemic.
  *In my case*,* it wasn’t possible to register online. Well*,* in Krakow*,* it’s possible there*,* but in smaller cities it’s more difficult*,* so that’s probably why. Because yes*,* it seems to me that it would be much easier to register online to see a doctor. (MWoj_4_30/12/2023*,* Item 145–146)*
c Motivation to use e-mail to contact a doctor
  Interviewees reported that they also used email to contact their doctor. The most common reason for this contact was to send test results. Interviewees appreciated this as a quick and paperless mode of communication.
  *Researcher: And have you ever used the option to email your test results to your doctor?*
  *Interviewee: Yes, I have sent them.*
  *Researcher: And how would you rate this experience?*
  *Interviewee: Very good. I didn't waste any time. The doctor replied quickly by e-mail, so I'm very satisfied.  (PSm_5_28.12.2023, points 368-375)*
d Slow adoption of the IPA
  Although the IPA portal has functions interviewees perceive as useful (see Theme 3), it is not used as frequently as expected. The following reasons were indicated:
  lack of knowledge (interviewees did not know that such an application exists or that it can be useful for them):
    *I haven't heard about it. (PSm_4_27/12/2023, Item 213)*)
  uselessness (the respondent rarely gets sick, does not have chronic diseases, and does not need to track their history or receive an e-prescription code, and therefore, there is no need to check it in the IPA):
    *I didn't actually use it. I know it exists, but I have never needed it for anything. Therefore, I haven’t used it. (EB_2_25/11/2023, Item 196)*
  lack of trust/fear of fraud
    Most frequently, interviewees expressed a lack of trust in the IPA website being secure against hackers:
    *When logging into such systems, you provide a lot of information about yourself and your health, and the risk is that someone may simply steal this data. (EB_5_8/12/2023, Item 346)*
    Most interviewees who use the IPA state that it is easy and convenient for them. They especially appreciated that the IPA allows them to track their or their children’s medical records.
    *Well, it's convenient, you don't have to carry a ton of paperwork with you, if you want to register with a doctor, all you need is a phone call. (EB_4_06/12/2023, Item 259)*
    It seems that the most common use of the IPA was to acquire a certificate of vaccination against COVID-19. For some interviewees, this was the only function they used. On the other hand, similarly to other solutions popularized during the pandemic, the IPA also turned out to be useful later for some interviewees.
e Other e-health applications
  Interviewees sometimes indicated other, less popular e-health applications. This is notable because the interviewees, who are not fully familiar with the meaning of the term 'e-health', are nonetheless looking for various solutions to facilitate their medical processes.
  Interviewee*: I sent the results to this application, which I said before, well because it's a condition to get reimbursement then I had to send something like this, it's a scan to the application "TU Health" that's what the application is called.  (PSm_9_28.12.2023, item 272)*

### Theme 2: barriers

A Internal
  This category refers to the functionality and user-friendliness of the application. Respondents indicated that some e-health applications were inconvenient, the login method was complicated, or the interface was not intuitive.
  a Skills
    Interviewees most often referred to their actual lack of skills, e.g., low internet literacy or insecurity when using e-health applications. Some of them had learned how to use e-health applications, but had not tried to use them yet.
*Researcher: What is the biggest problem for you when it comes to these healthcare applications?*
*Interviewee: It doesn't deter me, but I'm not convinced about these applications.*
*Researcher: And why?*
*Interviewee: Well, I didn't use it, maybe if I had to use it, I would gain some faith in it and I have such a skeptical approach to it. (PSm_9_28/12/2023, Pos. 237-240)*
*But in general, I am not able to deal with such applications. (PSm_7_28/12/2023, Item 225)*
    At this point, it is worth mentioning that age was seen to be a barrier to other people using e-health applications. Some participants emphasized that e-health can be more difficult for older people than it is for younger people because of different experiences with technology:
*Interviewee: It certainly would be easier for me [to register for a MD visit via the internet]. But when it comes to some older people, it would certainly be problematic. You need to know how to use this application, the internet, have a smartphone or a computer. So for me it would definitely be faster, because it's just a matter of choosing a date and then going to that doctor. You don't even have to talk or call there anymore. But I think that for some older people, it would definitely be problematic.*
*Researcher: So you feel prepared to use such services, right?*
*Interviewee: I think definitely.*
*Researcher: Mhm, so for you it's more about older people who have less contact with new technologies, right?*
* Interviewee: Exactly. rather, these programs are very intuitive now, so I think it wouldn't be a problem, but as I say, it depends on the age. (UZ_4_28/12/2023, Item 134-144)*
    Some interviewees said that their internet skills are lower than their children's, but they see this as a rather insignificant obstacle because they can count on children’s help. One of the older interviewees (62 y.o.) said:
     *I mean, at this point, I'm not prepared [to use e-health technology]. But if there was a need, then I think I would learn and use it. (UZ_7_29/12/2023, Item 128)*
  b Attitudes toward technology
    This subtheme encompasses barriers resulting from interviewees' attitudes toward technology. Most often, they feared data theft, technology addiction, or the unreliability of digital systems.
*It seems to me that at the same time, it is a great thing for humanity, but on the other hand, it is a threat, a huge threat because even my entire generation and generations younger than me are already addicted to phones, and it is absolutely obvious to us that without this, we are unable to function. We are overstimulated by all the social media, by this new technology. (MWoj_3_29/12/2023, Item 36)*
B External
  This subtheme concerns factors other than the motivations or skills of the users themselves. This is a relatively small group of factors in the analyzed material, but it deserves a more detailed description because it indicates problems often omitted in research. These are primarily
  a Limitations related to internet access, e.g., poor connection or the lack of a provider:
*From my recent visits to, for example, a family doctor, I conclude that the visit lasts 10 minutes, and it takes 15 minutes to enter data into the system, which freezes. In addition, there was a fight with a printer that did not print the prescription. (EB_6_12/12/2023, Item 437)*
  b Residing in small towns
    In more remote areas, healthcare facilities may rely only on direct patient contact and not offer remote services.
*Researcher: Do you register with a doctor online?*
*Interviewee: This is not possible in my clinic. (EB_2_25/11/2023, Item 202-204)*
*Well, for example, lack of access to the internet at some point when I was in some, I don't know, places far from some large urban center, where there is no reach, no internet. (MWoj_3_29/12/2023, Item 216)*

### Theme 3: facilitators

A) The possibility of saving time and reducing costs
  This is one of the main advantages interviewees mentioned of using e-health solutions. Examples they provided included avoiding queues to the clinic to make an appointment or making it easier to receive notifications about an upcoming visit, thus reducing the risk of mistakes in attending planned visits.
*I think it would change something about the waiting time because we are usually scheduled for specific hours, or at least for some span of time and so I would not waste time getting to the doctor. Well, and waiting with sick patients who go straight to the doctor. (MK_1_8.10.23, point 330)*
B) Everything in one place
  Regarding medical records, interviewees appreciated that one application, such as the IPA portal, may give them access to all their medical data. They also mentioned the reduced risk of losing some important results or referrals. Additionally, they valued the ability to manage their and their family's health. Parents especially saw the benefits of accessing their children’s IPAs and having their medical records at hand.
*First, I would have everything in one place. And whatever the need, quick and easy access to all of my medical records. (EB_2_25.11.2023, point 404)*
  Interviewees also liked the idea of accessing medical records online at a clinic or other medical facility.
*When it comes to paperwork like this, it can often get lost or misplaced somewhere. If I could have it in one place, say in a file, or even in several files, but in one place, in one application, I think that would be a great benefit to everyone. (UZ_4_28.12.2023, item 174)*
  Particularly, young parents prized the possibility of connecting their child’s IPA with their own. They appreciated having both records in one place.
*Well, for example, I would like to connect my child’s account to my own so that they could use this profile as well.  (PSm_5_28.12.2023, point 259)*
C) Zeitgeist
  The view that the development of technology is inevitable, and e-health is one manifestation of this, can also be interpreted as a facilitator of learning and using e-health applications.
*In general, I think that as the years go by, and let's keep it that way, technology will replace the human mind. (PSm_9_28.12.2023, item 58)*
*We all go, it all goes forward, with the times. That's what I said, there used to be phones like that, and now there are phones like this. Technology is advancing so much that... (MK_9_11.12.23, item 244)*

### Theme 4: willingness to share medical data

Most interviewees, including those with ambivalent attitudes toward e-health, stated that they would share their data for scientific purposes. They believed creating new medications or developing efficient treatment plans was important.:

a Interviewees with multimorbidities
  Interviewees with multimorbidities were willing to provide their medical data for scientific research and also expressed their willingness to monitor glycemia and blood pressure levels.
  *Yes, of course, it is very good for diabetes or blood pressure.  (PSm_7_28.12.2023, point 209)*
b Interviewees without health problems
  Research participants who did not report health problems at the time also expressed their willingness to support scientific research.
  *I am open-minded and on the issue of organ harvesting and on the issue of data donation, if you can help advance science, then absolutely yes. I really wanted to be a blood donor once, but they wouldn’t accept my blood. The reason is that I lost my eyesight due to eye cancer. Supposedly no documents, supposedly no tests, but as a result of the interview, or just in case, so as not to multiply bad genes, I understand. But mentally I'm all for it, and where I can be of use, there you go.  (MK_8_06.12.23, point 137)*
c Reasons for refusal to share medical data
  If interviewees were unwilling to consent to share medical data, the reason was their fear of personal data being stolen and used for previously unspecified purposes.
  *Because of security. Well, like I said, there's different people out there, let's just call them that, these hackers or something, that they find out that I'm this person and I give it to another person out there and they use my illness for example for disreputable purposes or something. I don't agree with that.  (MK_9_11.12.23, point 228)*

## Discussion

Qualitative analysis does not allow for drawing conclusions about population trends. However, selecting interviewees with diverse socio-demographic characteristics does make it possible to capture differences in the views and attitudes that may be observed in quantitative studies in larger study groups. Moreover, the answers to open questions offer insights that can guide planned quantitative studies. Our analysis revealed determinants of using e-health that can be facilitators and barriers.

### Theme 1: knowledge

#### Familiarity

The knowledge of the term ‘e-health’ was very low among the interviewees. This finding was also confirmed by other authors [19]. It seems that the term telemedicine is more recognized in the general population. Some interviewees believe that these two terms have the same meaning. Furthermore, low recognition of the term ‘e-health’ does not mean that interviewees do not use ehealth solutions. The fact that users do not know professional terms does not seemingly preclude their use of specific applications. Some of the interviewees used the IPA application, but they did not recognize its official name. They needed hints to confirm that they were IPA users, e.g., asking about printing the vaccine certificate from the governmental website. Relatively low knowledge about ehealth applications resulted from rare use. Sporadic users did not remember the applications’ names. In the case of the IPA application, its popularity declined after the COVID-19 pandemic, and people stopped logging into it. Finally, there is always the risk that participants of in-depth interviews do not share attitudes and have other experiences than those revealed by quantitative studies on large samples.

#### Experience

Interviewees asked about their experience with e-health most commonly spoke about eprescriptions, e-referrals, and electronic appointments for visits to their doctor. These types of e-health services are also popular in other countries [20]. Due to the introduction of the relevant Act, E-prescriptions became mandatory in January 2020 [3, 21]. Currently, referrals may be issued in the Polish healthcare system in both paper and electronic forms. There is also no legal obligation to enable patients to make appointments for visits to medical facilities electronically. The interviewed persons had a positive attitude toward electronic appointments and referrals, but the accessibility to those services depended on the capacities of the medical facilities they wanted to attend. It is worth mentioning that diversified e-solutions are more common in the private sector. Typically, private clinics offer their subscribers applications and web platforms that enable them to make appointments and contact doctors [22]. Nevertheless, the IPA, which is not assigned to any particular company and covers services related to public health insurance, has the greatest number of users.

Interviewees indicated the COVID-19 pandemic as an important factor in promoting the use of e-health applications. It seems that the wide use of ehealth solutions resulted from recommendations announced by the Ministry of Health and the NHF. The recommendations were an element of media advertising campaigns, e.g., about the IPA platform. Apart from these efforts, an important factor supporting the dissemination of e-health services was that interviewees valued the fact that ehealth services are safer and more convenient than traditional ones. As a result, they were also more likely to use them after the pandemic.

As mentioned earlier, some interviewees were unfamiliar with the IPA portal or believed it was not useful. The demarcation line between those who used and valued the IPA and those who did not seem to be determined by actual needs. Those who visited doctors rather rarely and needed prescriptions or referrals only occasionally did not consider the application useful [23–25]. On the contrary, interviewees who said that they visited doctors relatively often or regularly, as well as parents of minor children, appreciated that they could access medical history and related documents in one place [26]. Another factor influencing readiness to use the IPA was connected with interviewees’ trust in the cyber security of their personal data. This remains in agreement with previous research, which showed that the fear of personal or medical data theft could effectively discourage potential users from using IPA-like portals [27, 28].

### Theme 2: barriers

#### Internal barriers

Interviewees who stated that navigating e-health applications is too difficult also noted that, in general, they experience problems with new technologies. Such an association had been reported earlier [19]. Only one participant claimed that the IPA’s interface is not user-friendly. The lack of digital skills and the IPA’s usability were the main barriers.

Even interviewees who expressed a willingness to use new health technologies admitted that they feared having their personal data stolen, which has already been mentioned regarding the IPA. Other authors also observed that the fear of exposing personal data was a frequently cited barrier to accessing e-health technologies [29, 30]. Interestingly, younger participants often mentioned age as a barrier to using e-health applications. However, older persons mostly expressed interest in new technologies. We have not observed that older age predicts reluctance to use e-health solutions. However, it should be noted that in-depth interviews do not allow for a reliable assessment of general trends in society. Apart from this, in our sample, there were only five persons at least 60 years old, so assessing the attitudes toward e-health in older age groups of society was hardly possible.

Due to the continuous development of technology and the frequent replacement of traditional healthcare encounters with digital ones, participants are also afraid of dependence on technology. It is noteworthy that despite the abovementioned concerns, interviewees had used at least one e-health solution for a longer time. It was a common finding that if they had problems coping with e-health, they asked relatives for help.

#### External barriers

Although access to the internet does not seem to be an obstacle even for residents of remote areas, some participants complained of technical problems. In general, they reported poor quality of internet access, manifesting in unstable connections, long loading times for webpages, and too much time required for internet searches or even logging in to the application. All these problems significantly influenced their willingness to use e-health solutions.

The residents of remote areas also talked about undeveloped e-health services in the healthcare institutions available there. For example, the only option for making an appointment in a clinic was frequently telephone contact. Simultaneously, they rather rarely used the IPA, and some were even unaware of it.

Finally, we should add that for some of the study participants, the fear of losing internet connection during a health emergency was a deterrent to using e-health services.

### Theme 3: facilitators

#### Time-saving and the reduction of expenditures

Most interviewees said they have many responsibilities and obligations, and saving time is a key motivation for using e-health. Some of them acknowledged that due to being in a hurry, they had made mistakes in writing down the date or time of an appointment and had made unnecessary trips to medical clinics. Earlier studies have shown that patients appreciate reminder calls, SMSs, or e-mails [31]. Most medical facilities can already send appointment confirmations using various communication channels, greatly improving the flow of scheduled appointments [32–34].

#### “Everything is in one place”

The interviewees greatly appreciated access to the repository containing the documentation of previous medical encounters, ordered medication and other health-related information. In this context, the IPA portal was found to be useful and convenient.

It seems that two opinions competed among study participants. One group believed there was no need for full digitalization of medical records and preferred to have them printed. Such an attitude is probably rooted in a distrust of e-health solutions or technology. The interviewees from this group claimed they rarely needed such records, so they do not need any special application or account to keep them. The second group included advocates of online patient accounts. They argued that using the IPA ensures that they never lose documentation. Furthermore, sending it to a doctor via an ehealth platform or even by e-mail is more convenient than bringing doctors “tons of printed stuff.” Finally, they believed that access to medical records during visits or a stay in a healthcare facility greatly improves the treatment process. Such areas of encouragement were reported by both people struggling with multimorbidity and those who assessed their health as good.

#### Zeitgeist

Some interview participants believed that using e-health applications is inevitable as digitalization progresses in all areas of life and that it cannot be avoided. Several of those interviewees showed an ambiguous attitude toward new technologies, while others perceived it as a favorable aspect of technological development. The views of the first subgroup were puzzling as apparently their skepticism toward technology was overcome by their faith in the irreversibility of general trends like technological progress and innovation. The statements about the inevitability of such progress may be treated as a kind of social (or technological) conformism [35].

#### Theme 4: willingness to share medical data

The interviewees showed a positive attitude to sharing their medical data for research purposes. Their health status and the presence of medical conditions did not matter in this regard. Interviewees with multiple diseases accepted data sharing to a similar degree as those in good health. No significant differences were seen which depended on socio-demographic features, as confirmed by other researchers [36–38]. The main argument articulated by the interviewees was the willingness to improve the treatment of people with similar medical conditions. A higher acceptance of using e-health applications was a by-product of such an attitude, as interviewees perceived digitalization as a way to access medical data. However, in this context, they focused on e-health solutions provided by public (governmental) institutions, e.g., the IPA portal. Evidently, the interviewees underlined the non-commercial use of their medical data, and according to them, such use could only be ensured by public institutions.

The interviewees who were unwilling to share their medical data were afraid of issues with data security and possible fraud, e.g., the use of data for purposes other than those initially declared. It seems that governmental institutions in other countries are also perceived as trustworthy and secure [39]. Another source of skepticism was the general perception of e-health applications and other digital solutions as a threat to privacy.

### Limitations

The qualitative method does not allow conclusions to be drawn about the whole population. However, it allows one to know and understand interviewees’ attitudes and motivations for their decisions, as well as their interpretations. Another characteristic of qualitative research, non-probability sampling, also prevents the generalization of obtained results. Moreover, there is a possibility that people with specific attitudes will predominate among the interviewees. This cannot always be avoided during recruitment and may lead to selection bias [36]. In the case of our study, there was a risk that people with prevailing positive attitudes toward e-health would be more inclined to join the study.

## Conclusions

Our study confirmed that using concrete solutions does not suggest a full understanding of the ehealth domain. Insufficient skills and negative attitudes toward technology still hinder the adequate adoption of e-health applications. Although the penetration of internet access has increased significantly during the last decade in Poland, still, in remote areas, the insufficient quality of the connection may be a deterrent to using e-health. Furthermore, healthcare institutions in such areas are less prone to adopt e-services for their patients.

The main facilitators to citizens using e-health solutions include the perceived benefits in terms of time savings and decreased expenditures to obtain traditional services. End-users also appreciate specific features of the available e-health applications. In the case of the IPA, one such positive aspect was the ability to access the previous medical records in one digital repository and share them with healthcare providers. The acceptance of e-health use was also associated with a positive attitude toward sharing personal data for the benefit of other patients.

## Supplementary Information

Supplementary Material 1.

## Data Availability

The dataset generated and analyzed during the current study is available in the ZENODO repository (10.5281/zenodo.11046955) [40].
