# Supplementary material for: The perception of facilitators and barriers to the use of e-health solutions in Poland: a qualitative study
Source: BMC Med Inform Decis Mak. 2024 Dec 18;24:381. doi: 10.1186/s12911-024-02791-x (PMC11653833; doi:10.1186/s12911-024-02791-x)
Supplement: Supplementary file 1 — Supplementary Material 1. [file 12911_2024_2791_MOESM1_ESM.pdf]

Table S1. The Structure of the Interview Guide

1)	Health needs and the utilization of healthcare j
2)	The attitudes to new technologies and technology anxiety
3)	The use of the Internet to access health-related information
4)	Contacts with other persons/patients with similar medical conditions via the Internet and social media
5)	The knowledge and understanding of telemedicine and e-health
6)	The utilization of e-health applications
7)	The use of health apps on mobile phone. The need for the use of health monitoring applications
8)	The use of e-health applications available for citizens in Poland, e.g., Internet Patient Account
9)	Experience with remote physician visits.
10)	Barriers to the use of e-health applications. Main sources of anxiety related to e-health
11)	Readiness to use remote care in the future
12)	The use of other digital services apart from e-health
13)	Self-assessed e-health literacy
14)	The perception of digital skills of the health professionals
15)	Access to electronic medical record
16)	The perception of sharing own medical data for research
17)	The attitudes to the use of AI in healthcare
18)	The attitude to the use of robots in healthcare

Table S2. Code Tree

Code	Frequency
Code tree	339
IPA: Internet patient account	
motivation to use IPA	
faster access to specialists	1
appointment reminder	1
If unavoidable	2
Comfort	7
Does not use IPA	20
Use IPA	18
Use of apps	5
Gov apps	13
E-health apps	30
Healthy lifestyle apps	8
shopping online	17
Does not use health apps	12
Banking	28
Health monitoring	0
cons: afraid of data fraud	2
pros	8
Technologis in med: pros	
Positive attiude	10
Neutral - obsever (+)	1
Ambivalent	2
incomparable	2
Time-saving	10
Convenient	37
Does not feel prepared but is ready to use	1
Fast changes	1
No difference	3
No risk of losing the documents (+)	3
Technologies in med: barriers	2
Not useful for him/her	16

Login is complicated	2
Inefficient	1
problems with equipement/apps	2
lack of digital literacy	4
small town	1
age related	9
technology takes a lot of time	1
afraid about personal data	6
too many technologies (+)	3
Risk of becoming addicted to	3
Banking is more developed	2
knowledge of the term e-health	6
no	23
yes	9
E-health - apps types	7
